# *GAS1* Deficient Enhances UPR Activity in* Saccharomyces cerevisiae*

**DOI:** 10.1155/2019/1238581

**Published:** 2019-06-02

**Authors:** Hong-jing Cui, Xin-gang Cui, Xia Jing, Yuan Yuan, Ya-qin Chen, Ya-xin Sun, Wei Zhao, Xin-guang Liu

**Affiliations:** ^1^Guangdong Provincial Key Laboratory of Medical Molecular Diagnostics, Institute of Aging Research, Guangdong Medical University, Dongguan 523808, China; ^2^Mudanjiang Medical College, Mudanjiang 157011, China; ^3^Reproductive Medicine Center, Affiliated Hospital of Guangdong Medical University, Zhanjiang 524023, China

## Abstract

Beta-1,3-glucanosyltransferase (Gas1p) plays important roles in cell wall biosynthesis and morphogenesis and has been implicated in DNA damage responses and cell cycle regulation in fungi. Yeast Gas1p has also been reported to participate in endoplasmic reticulum (ER) stress responses. However, the precise roles and molecular mechanisms through which Gas1p affects these responses have yet to be elucidated. In this study, we constructed* GAS1-*deficient (*gas1Δ*) and* GAS1*-overexpressing (*GAS1 OE*) yeast strains and observed that the* gas1Δ *strain exhibited a decreased proliferation ability and a shorter replicative lifespan (RLS), as well as enhanced activity of the unfolded protein response (UPR) in the absence of stress. However, under the high-tunicamycin-concentration (an ER stress-inducing agent; 1.0 *μ*g/mL) stress, the* gas1Δ *yeast cells exhibited an increased proliferation ability compared with the wild-type yeast strain. In addition, our findings demonstrated that* IRE1* and* HAC1 *(two upstream modulators of the UPR) are required for the survival of* gas1Δ* yeast cells under the tunicamycin stress. On the other hand, we provided evidence that the* GAS1 *overexpression caused an obvious sensitivity to the low-tunicamycin-concentration (0.25 *μ*g/mL). Collectively, our results indicate that Gas1p plays an important role in the ageing and ER stress responses in yeast.

## 1. Introduction

Newly synthesized secretory and transmembrane proteins fold and assemble in the endoplasmic reticulum (ER) with the assistance of chaperones, oxidative folding components, and other systems that support protein posttranslational regulation. Impairments in this complex process cause the accumulation of unfolded/misfolded proteins, provoking a cytoprotective signalling cascade termed the unfolded protein response (UPR) [1]. In mammalian cells, the UPR signalling pathway and ER protein homeostasis are regulated by three known transmembrane ER stress sensors or transducers: inositol-requiring protein 1 (IRE1), double-stranded RNA-activated protein kinase-like ER kinase (PERK), and activating transcription factor 6 (ATF6). However, in the relatively simple organism* Saccharomyces cerevisiae*, the UPR signalling pathway is primarily mediated by the ER stress-sensing factor (*IRE1*) and the transcription factor homologous to ATF/CREB 1 (*HAC1*) [2]. Activation of the UPR signalling pathway enhances the ability of the ER to deal with the accumulation of unfolded/misfolded proteins, relieves the ER stress damage, and maintains ER protein homeostasis [3, 4].

The cell wall of the budding yeast* S*.* cerevisiae *can protect cells from various stresses, such as osmotic pressure changes and noxious chemicals. An elastic cell wall is supramolecular structure consisting of four primary components (beta-1,3-glucan, beta-1,6-glucan, chitin, and mannoproteins) [5]. The formation and maintenance of beta-1,3-glucan chains is primarily catalysed by beta-1,3-glucanosyltransferase (Gas1p), which is initially characterized at the cell wall via a glycosylphosphatidylinositol (GPI) anchor and also localizes to the nuclear periphery. Gas1p has been reported to play an important role in cell wall biosynthesis and transcriptional silencing in yeast. For example, the loss of Gas1p activity results in swollen, more spherical cells and causes defects in cell wall assembly that decrease cell viability and enhance cell sensitivity to heat and cell wall-disrupting compounds such as Congo red [6]. On the other hand, Gas1 deficiency results in broad DNA damage sensitivity and defects in telomeric silencing, which is involved in regulating senescence [7, 8]. Therefore, the different locations of Gas1p in yeast cells should determine its diverse functions.

Gas1p disruption has been reported to increase the resistance to the ER stress-inducing agents such as tunicamycin (Tm) in a large-scale survey [9]. In this study, we attempted to further explore the precise roles of Gas1p disruption on ER stress responses and elucidate the underlying molecular mechanisms in* S*.* cerevisiae*. Our results show that a* GAS1 *deficiency* S*.* cerevisiae* strain exhibited a decreased proliferation ability and a short-lived replicative lifespan (RLS), as well as an enhanced UPR activity in the absence of Tm-induced stress. However, the difference in proliferation rates between the* gas1Δ* and wild-type yeast strains decreased with the increasing concentrations of Tm, and especially under high-Tm-concentration (1.0 *μ*g/mL) stress, the* gas1Δ* yeast cells exhibited an increased proliferation ability compared with the wild-type yeast strain. In addition, our findings demonstrated that* IRE1* and* HAC1 *(two upstream modulators of the UPR) were required for the survival of* gas1Δ *yeast cells under the Tm stress. On the other hand, we demonstrated that the* GAS1 *overexpression had no influence on the lifespan of yeast cells with no increase in UPR activity (under the unstressed condition) and caused a notable sensitivity to Tm (0.25 *μ*g/mL). These findings highlight the important roles of Gas1p in the ageing and ER stress responses in yeast cells.

## 2. Materials and Methods

### 2.1. Strains and Plasmids

The wild-type yeast strain (BY4741 and BY4742) and the plasmid pRS305 were gifts from Dr. Matt Kaeberlein (University of Washington, USA). The strains used in this study were derived from BY4741 (MATa* his3Δ1 leu2Δ0 met15Δ0 ura3Δ0*) and BY4742 (MAT*α his3Δ1 leu2Δ0 lys2Δ0 ura3Δ0*) (Table 1). The strain lacking* GAS1 *(BY4742* gas1::LEU2*;* gas1Δ*) was produced through PCR-mediated one-step gene disruption (primers: 5′-TGATAAAACAAAAACAACAAACACAGCTAAATCTCA-ACAAGATTGTACTGAGAGTGCAC-3′ and 5′-CCATACCTTATCGAGTTATTATGTATGTGTCG-AAGCTTTCTGTGCGG-TATTTCACACCG-3′) using the plasmid pRS305 with* LEU2* as the selectable marker [10].

*GAS1* overexpression strains (*GAS1 OE*) were constructed by transforming a* pSh*AI linearized* GAS1*-overexpression plasmid into BY4742 yeast cells. The plasmid pRS305-*GAS1*-*OE *was constructed by inserting a 2598-bp* Bam*HI-*Sac*I fragment that was amplified from yeast genomic DNA into the corresponding sites in pRS305 (the primers used to construct the vectors are listed in Table S1). In addition to the* GAS1* ORF, ~609 and ~308 bp upstream and downstream of the* GAS1* ORF, respectively, were amplified such that* GAS1* expression would be driven by its natural promoter [11–13]. The* GAS1* deficient and overexpression strains were individually mated with the BY4741 strain, and after dissecting meiotic tetrads under an optical microscope, individuals cells were cultured until a single colony formed on YPD plates at 30°C.

The* GAS1 *and* IRE1 *or* HAC1 *double-deletion strains (*gas1Δire1Δ *and* gas1Δhac1Δ*) were constructed by transforming a* Mlu*I-digested plasmid (pRS305-*gas1*-ko) into the* IRE1* and* HAC1 *deletion strains (*ire1Δ* or* hac1Δ*) [10, 13]. First, the plasmid pRS305-*gas1*-ko was constructed to delete* GAS1*. Briefly, a PCR-amplified* Mlu*I-*Bam*HI fragment (nucleotides -500~0 bp) and a* Hin*dIII-*Mlu*I fragment (nucleotides 1681~2173 bp) were cloned into the* Hin*dIII-*Bam*HI sites of pRS305 (the primers used to construct the vectors are listed in Table S1). Approximately three-microgram DNA of the linearized pRS305-*gas1*-ko was transformed into the* ire1Δ* or* hac1Δ* strains, with the entire ORF of the target gene removed in each case via the homologous recombination.

The above strains were generated using a modified lithium acetate transformation method and were verified by PCR (the primers used to verify mutant strains are listed in Table S2, and Agarose gels of PCR products for verifying mutant strains are listed in Figures S1 and S1–S5).

### 2.2. Tunicamycin Resistance

The resistance of yeast cells to the ER stress inducer tunicamycin (BBI, TF1129) (0.125, 0.25, 0.5, and 1.0 *μ*g/mL Tm) was assessed by performing spot assays and growth curves determinations. For these assays, single colonies of each of studied yeast strains were inoculated into 3 mL liquid YPD medium and cultured overnight to the exponential phase (optical density (OD) of approximately ~2.0) with shaking at 180 rpm. Subsequently, the cultures were 10-fold serially diluted, and 4 *μ*L of each dilution was spotted onto standard YPD plates that was then incubated at 30°C until colonies formed, with images taken every 12 h.

The growth rates of the yeast strains were assayed using a Bioscreen C instrument (Growth Curves, USA) [14–16]. The working volume of the yeast cultures supplemented with or without Tm was 300 *μ*L (OD600~0.04) in the wells of the cell culture plates. The strains were cultured at 30°C with shaking at 180 rpm for 2 days, and OD of cultures was measured automatically at 600 nm at regular 2 h intervals. For each type of culture medium, the average absorbance values of three cultures grown for 48 h and performed induplicate were used to generate growth curves for each studied strain. All of the results were calculated using the Friedman Test with a significance level of 5%.

### 2.3. UPR Activity

UPR activity was evaluated by analysing the* HAC1* transcript splicing, the transcription of canonical UPR target genes, and the levels of Kar2p protein. For these assays, total RNA was harvested from yeast strains treated with or without 1 *μ*g/mL Tm for 1 h and reverse transcribed following the manufacturer's instructions (TaKaRa). To analyse* HAC1* mRNA splicing, the primers used to PCR amplify* HAC1* cDNA were HAC1-F (CCGTAGACAACAACAATTTG) and HAC1-R (CATGAAGTGATGAAGAAATC) [17]. PCR fragments were electrophoresed in 2% (w/v) agarose gels stained with Goldview and quantified by densitometry using Image J.

The transcription of canonical UPR target genes (*EUG1*,* ERO1*,* FKB2*,* LHS1*,* PDI1*, and* KAR2*) was assessed by quantitative real-time PCR as previously described (the primers used for real time qPCR are listed in Table 2) using the housekeeping gene* PRP8* and the comparative Ct method to determine the abundance of each gene [13, 18]. The experiments were independently repeated six times using at least three samples. The data was evaluated using Student's* t*-test, with* p *< 0.05 and* p *< 0.01 indicating significant and highly significant differences, respectively.

Total yeast protein samples were derived from cultures grown for 12 h and were separated by 10% SDS-PAGE and subsequently electrotransferred to PVDF membranes by semidry transfer. The visualized immunoreactive bands with the fluorescent signals were analysed using a western blot imaging system (Azure c400). The relative expression levels of Kar2p in the BY4742,* gas1Δ*, and* GAS1 OE *strains were evaluated using the polyclonal goat anti-rabbit antibodies (Kar2p, Santa Cruz, sc33630; G6PDH, Sigma Aldrich, A9521).

### 2.4. Replicative Lifespan

The replicative lifespan (RLS) of the strains was measured as previously described for the BY4742,* gas1Δ*, and* GAS1OE *strains [13, 19]. All of the strains were streaked onto standard YPD plates and grown at 30°C for 2 days. Subsequently, single colonies were plated onto fresh YPD plates and grown for 1 day. Yeast cells used for RLS analysis were plated onto fresh YPD plates for overnight growth. Each experiment utilized approximately 20 cells per genotype and was independently measured at least five times. Buds from yeast mother cells were physically separated using a manual micromanipulator under an optical microscope (Axio Scope A1, Carl Zeiss, Gottingen, Germany), and mother cells were allowed to undergo one to two divisions. RLS data were analysed using a two-tailed Wilcoxon rank-sum test with Wilcoxon* p*-values of* p *< 0.05 and* p *< 0.01 indicating significant and highly significant differences, respectively.

## 3. Results and Discussion

### 3.1. GAS1 Deficiency Shortens the Replicative Lifespan of Yeast Cells

**Figure 1:**
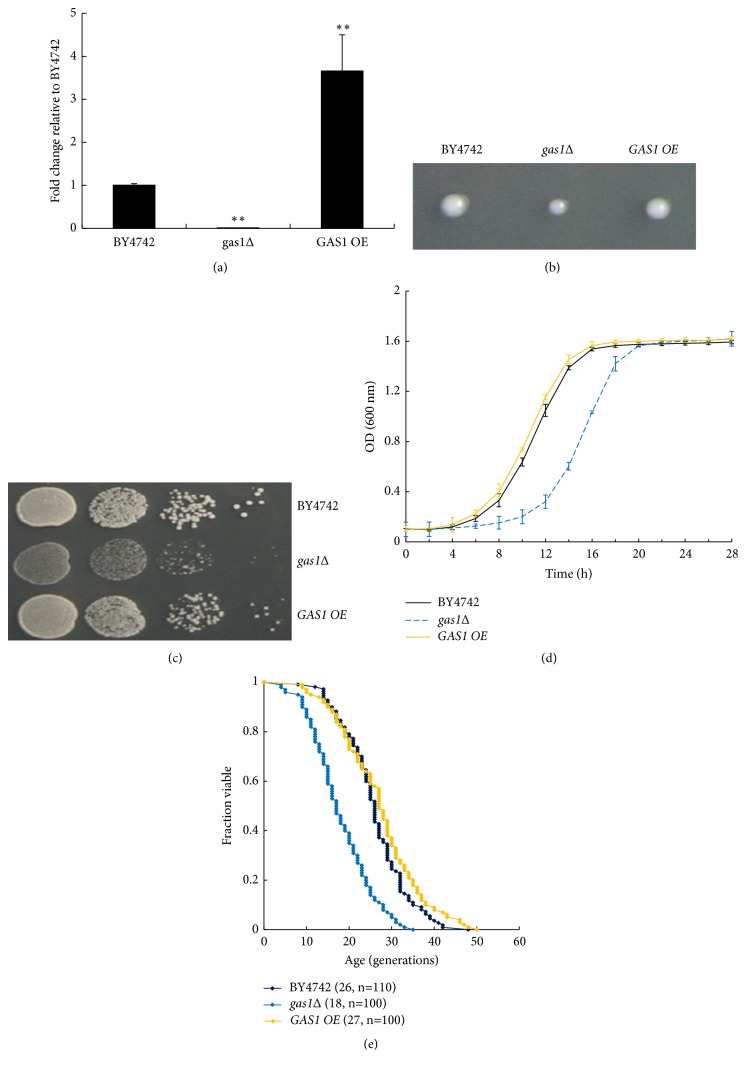
*GAS1* deficiency decreases the growth rates and RLS of yeast cells. (a) Relative transcription level of* GAS1* mRNA in the* GAS1 *deficient (*gas1Δ*) and* GAS1* overexpression (*GAS1 OE*) strains. The data are expressed as the means ± SD (n=3). *∗∗p* < 0.01* vs.* BY4742. (b) Spore progenies were grown into colonies from single cells on YPD plates after backcrossing. (c) Yeast cells were serially diluted (1:10) and cultured on YPD plates. (d) Growths of the BY4742,* gas1Δ*, and* GAS1 OE* strains were monitored (OD_600_) at various time points. (e) RLS curves of the BY4742,* gas1Δ*, and* GAS1 OE* strains. Mortality curves were generated from lifespan data, and BY4742 was considered to be the control. Numbers in parentheses are the mean RLS and cell number values.

To confirm the role of yeast Gas1p in cell proliferation, firstly,* GAS1*-deficient (*gas1Δ*) and* GAS1*-overexpressing (*GAS1 OE*) strains were first constructed and verified by quantitative RT-PCR. The* GAS1 OE* strain displayed a roughly threefold higher expression level of* GAS1* mRNA than the wild-type yeast (BY4742) strain (Figure 1(a)). Subsequently, the results observed for colonies from single cells (Figure 1(b)) and spot assays (Figure 1(c)) demonstrated that the* gas1Δ *strain displayed smaller colonies than the BY4742 strain, and the* GAS1 OE *strain exhibited a normal growth as the BY4742 strains.

The division of* S. cerevisiae* cells is asymmetric, because a mother cell always produces a smaller daughter cell during each division, with the total number of daughter cells produced before a mother cell dies being greater when the replicative lifespan (RLS) of the mother cell is longer. To assess the budding ability of the yeast cells under the physiological conditions, we assessed the growth rates of strains using the Bioscreen C MB system and determined the RLSs of the* gas1Δ* and* GAS1 OE* strains under an optical microscope. Consistent with the above results, the* gas1Δ *strain grew more slowly than the* GAS1 OE* and BY4742 strains, with* GAS1* deficiency reducing the RLS of yeast cells by approximately 35% (*p* < 0.01) (Figures 1(d) and 1(e)). In addition,* GAS1* overexpression did not exert any influence on the growth rates and RLS of yeast cells (Figures 1(d) and 1(e)). According to these results,* GAS1 *deficiency decreased the growth rates and shortened the lifespan of* S*.* cerevisiae *cells.

These results indicate that Gas1p is involved in regulating lifespan of wild-type yeast cells. One possibility is that Gas1p disruption causes defects in cell wall assembly and decreases the cell viability [6]. Another important possibility is that the unglycosylated proteins caused by Gas1p dysfunction should be accumulated, potentially impairing proteome maintenance, causing constitutively ER stress responses, and possibly resulting in UPR-mediated cell death. In addition, Gas1p deficiency causes broad DNA damage sensitivity and defective cell cycle checkpoint activation, which cause a phenotype resembling premature ageing [8, 20]. Consistent with this observation, Gas1p disruption has previously been shown to reduce growth rates by approximately 25%, while fivefold Gas1p overexpression was not observed to affect cell growth parameters and cell wall properties [21]. Furthermore, a* gas1Δ *strain of* Schizosaccharomyces pombe* has been observed to only grow in osmotically supported media [6]. Collectively, the above results suggested that* GAS1 *deficiency induces the ageing in yeast cells, but the specific mechanisms by which* GAS1 *deficiency regulates lifespan are still not clear.

### 3.2. GAS1 Deficiency Enhances the Activity of the UPR Signalling Pathway

**Figure 2:**
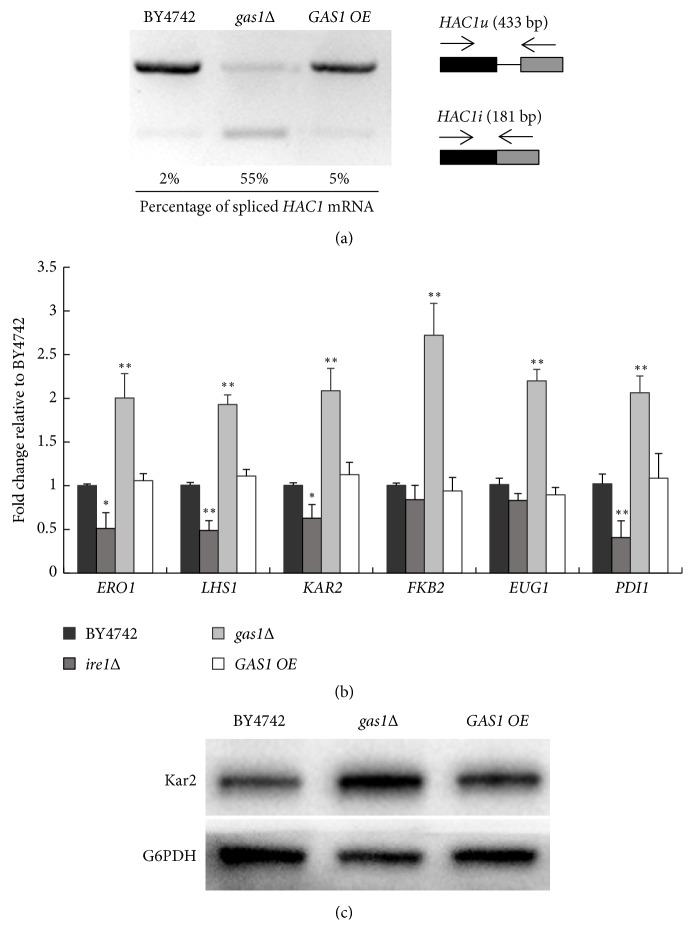
*GAS1* deficiency enhances the UPR activity in yeast cells. (a)* HAC1* mRNA splicing in the BY4742,* gas1Δ*, and* GAS1 OE *strains.* HAC1u *and* HAC1i* indicate the unspliced and spliced* HAC1* mRNA, respectively. (b) Relative expression of UPR target genes, including* ERO1*,* LHS1*,* KAR2*,* FKB2*,* EUG1*, and* PDI1* in the BY4742,* ire1Δ*,* gas1Δ*, and* GAS1 OE *strains. The data represent the means ± S.D. (*n* = 6). *∗ p *< 0.01* vs* BY4742; *∗∗ p *< 0.01* vs* BY4742. (c) Relative expression of Kar2p in the BY4742,* gas1Δ*, and* GAS1 OE* strains determined by western blotting, with G6PDH used as an internal control. BY4742 was considered to be the control.

The UPR pathway is one of the best-characterized pathways for alleviating ER stress, and* HAC1* mRNA splicing by the ER stress sensor Ire1p, which is an essential step in the activation of the UPR pathway, is commonly used to evaluate UPR activity [17]. Thus, we quantified the levels of spliced and unspliced* HAC1* mRNA in the BY4742,* gas1Δ*, and* GAS1 OE *strains. The results showed that the* gas1Δ *strain displayed increased levels of spliced* HAC1* mRNA (55±16%), relative to the BY4742 (2±0.8%) and* GAS1 OE* (5±1.5%) strains (Figure 2(a)). These data indicated that the* HAC1* mRNA splicing was induced in the* GAS1*-deficient yeast strain, suggesting the existence of ER stress in this strain.

We further evaluated the intracellular UPR activity by assessing the transcriptional levels of several canonical UPR target genes by quantitative RT-PCR in the BY4742,* gas1Δ*, and* GAS1 OE *strains. The results showed that the expressions of most of the UPR target genes, such as those involved in the oxidative folding (*EUG1*,* ERO1*, and* PDI1*), trafficking (*FKB2*), and chaperoning (*KAR2 *and* LHS1*) of proteins, were significantly higher in the* gas1Δ *strain than in the BY4742 strain. However, the expressions of most UPR target genes were not altered in the* GAS1 OE *strain compared with those of the BY4742 strain (Figure 2(b)). Consistent with the data of quantitative RT-PCR results, Kar2p protein levels were noticeably higher in the* gas1Δ *strain than in the* GAS1 OE *and BY4742 strains (Figure 2(c)). Based on these results, UPR activity was enhanced in the* GAS1*-deficient yeast strain but not in the* GAS1*-overexpressing yeast strain.

Previous reports have revealed that enhanced UPR activity is closely associated with lifespan in long-lived yeast, worm,* Drosophila*, and mammalian cell mutants [17, 22–24]. In this study,* GAS1*-deficient yeast cells displayed the enhanced intracellular UPR activity but shortened RLS, indicating that the UPR activity was not beneficial for longevity in the* GAS1*-deficient strain. One possible explanation for our observation is that mild or moderate ER stress stimulates an adaptive UPR to promote cellular survival whereas, in the case of a persistent and overwhelming stress, UPR accelerates the apoptotic cell death process [25].

Similar to our observations, the* Caenorhabditis elegans gas-1*(*fc21*) mutant also exhibited an upregulated mitochondrial UPR and a decreased lifespan due to an abnormal mitochondrial function [26]. In addition, muscle-specific Cisd2-knockout mice used as premature ageing models also exhibited selectively or strongly activated ER stress UPR pathways [27]. In our study, we extended these findings by demonstrating that the short-lived* GAS1*-deficient strain induced the activity of UPR pathway, indicating that the UPR activity is equally induced in both short- and long-lived mutants and may be therefore uncoupled from their longevity.

### 3.3. Proliferation Rates of the GAS1 Deficient and Overexpression Yeast Strains under the Tunicamycin-Induced Stress

**Figure 3:**
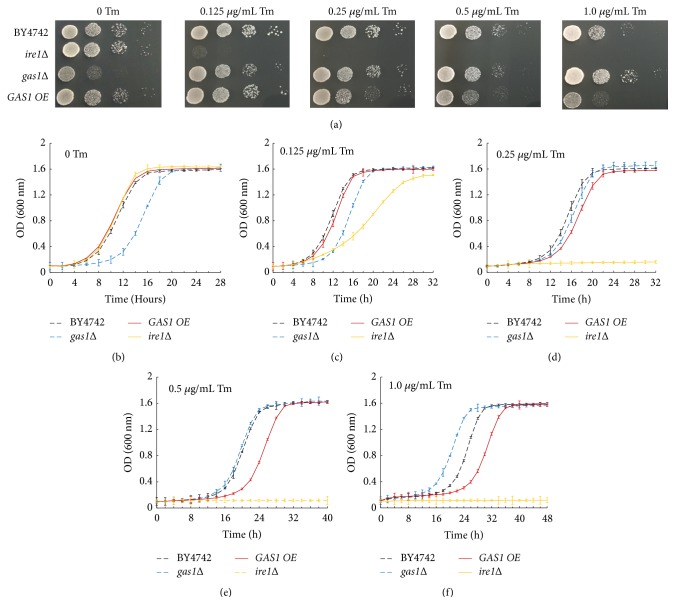
The proliferation rates of* GAS1* deficient and overexpressing strains under the tunicamycin-induced stress. (a) Yeast cells were serially diluted (1:10) and cultured on YPD plates with or without 0.125, 0.25, 0.5, or 1.0 *μ*g/mL Tm. (b)–(f) Growth curves of the BY4742,* ire1Δ*,* gas1Δ*, and* GAS1 OE* strains treated with or without 0.125, 0.25, 0.5, or 1.0 *μ*g/mL Tm were monitored (OD_600_) at various time points. Tm denotes Tunicamycin, *∗* represents a big difference, and BY4742 was considered to be the control.

Gas1p is often modified to a misfolded version and used as a model to explore the specific mechanisms of ER protein quality control [28]. To further study the role of Gas1p in ER stress responses, we observed the colony-forming abilities and growth curves of the* gas1∆* and* GAS1 OE* strains in the presence or absence of an ER stress-inducing agent (tunicamycin, Tm). The results showed that the difference in proliferation rates between the* gas1Δ *and BY4742 strains decreased under the low-Tm-concentration stress (0.125, 0.25, and 0.5 *μ*g/mL Tm) (Figures 3(a)–3(e)), whereas the* gas1∆* yeast cells grew faster than the BY4742 strain under the high-Tm-concentration stress (1.0 *μ*g/mL Tm) (Figures 3(a) and 3(f)). In addition, the* GAS1 OE *strain exhibited the normal growth, similar to that of the BY4742 strain, under the low-Tm-concentration stress (0.125 *μ*g/mL Tm) (Figures 3(a)–3(c)), as well as significant sensitivity under the high-Tm-concentration stress (0.25, 0.5, and 1.0 *μ*g/mL Tm) (Figures 3(a), 3(d)–3(f)). Thus, the results indicated that Gas1p is involved in regulating the ER stress responses.

We should note that the* gas1∆* strain displayed obviously the ER stress resistance under the high-Tm-concentration stress compared with the BY4742 strain. One possibility for this result is that the basal UPR activity induced by the* GAS1 *deficiency was beneficial for resisting the ER stress-induced injury. Another important possibility is that other protective signalling pathways were triggered by* GAS1 *deficiency, such as a set of cell wall compensatory mechanisms to maintain the cell integrity [29–31], ultimately leading to the resistance of* gas1∆ *strain under the high-Tm-concentration stress.

Our finding also demonstrated that* GAS1*-overexpressing yeast cells displayed significantly the ER stress sensitivity under the low-Tm-concentration stress. One possibility for this result is that Tm, as an N-linked glycosylation inhibitor, can block the glycosylation of the overexpressed Gas1 protein, resulting in the accumulation of misfolded protein within the ER and ultimately leading to cell death [32, 33]. Thus, our findings indicated that Gas1p is involved in regulating ER stress responses.

### 3.4. Deletion of IRE1 or HAC1 Impairs the Survival of a GAS1-Deficient Strain under the Tunicamycin-Induced Stress

**Figure 4:**
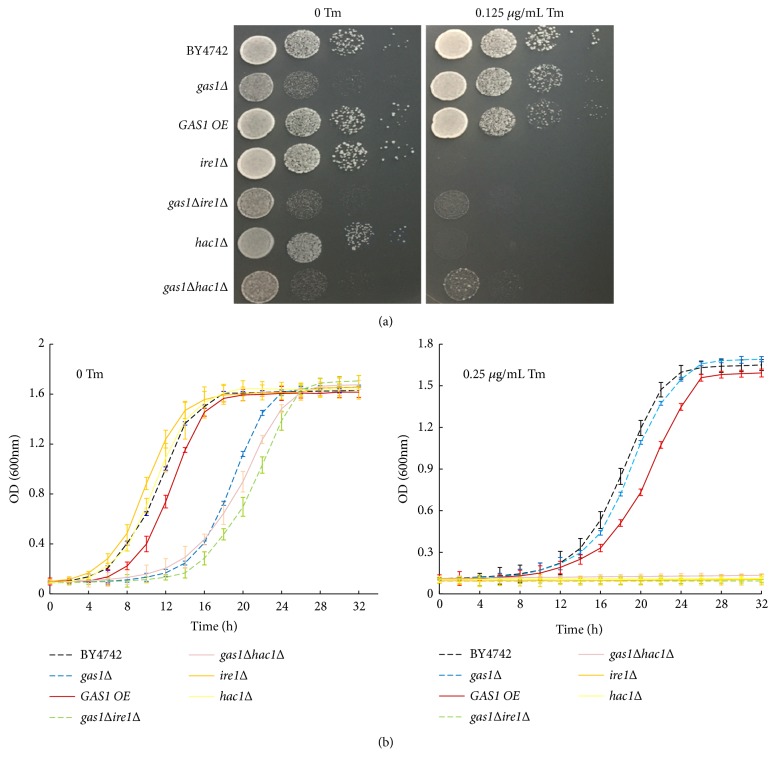
*IRE1* and* HAC1* are required for the survival of* gas1Δ *yeast cells under the tunicamycin-induced stress. (a) Yeast cells of the BY4742,* gas1Δ*,* GAS1 OE*,* ire1Δ*,* hac1Δ*,* gas1Δire1Δ*, and* gas1Δhac1Δ *strains were serially diluted (1:10) and cultured on YPD plates with or without 0.25 *μ*g/mL Tm. (b) Growth curves of the mutant strains treated with or without 0.25 *μ*g/mL Tm were monitored (OD_600_) at various time points. Tm denotes Tunicamycin, and BY4742 was considered to be the control.

To further explore the possible relationship between the growth of* gas1Δ *yeast cells and UPR activity, we constructed the double-gene deletion strains lacking either* IRE1 *or* HAC1 *in addition to* GAS1 *(*gas1Δire1Δ *and* gas1Δhac1Δ*, respectively). The* IRE1 *and* HAC1 *single-deletion strains (*ire1Δ *and* hac1Δ*) were not significantly affected with respect to their colony-forming abilities and proliferation rates under physiological conditions, as previously reported [17]. In the absence of Tm-induced stress, the* gas1Δ*,* gas1Δire1Δ*, and* gas1Δhac1Δ *strains grew slowly compared to the BY4742,* GAS1 OE*,* ire1Δ*, and* hac1Δ *strains (Figure 4). After Tm treatment, concurrent either* HAC1 *or* IRE1 *deficiency severely impaired the proliferation abilities of* gas1Δ *strain, suggesting that either* HAC1 *or* IRE1 *was required for the growth of* gas1Δ *yeast cells under the Tm stress (Figure 4). Thus, these data indicated that* HAC1 *and* IRE1* are required for the survival of* gas1Δ* strain under the Tm-inducing stress.

Ire1p and Hac1p, which are two upstream modulators of the best-characterized UPR pathway, are involved in regulating the ER stress responses in yeast cells. The cytosolic (or transmembrane) domain of Ire1p senses membrane aberrancy, the activated Ire1p interacts with the accumulated unfolded proteins in the ER and then splices the transcription factor* HAC1* mRNA, and ultimately the activated Hac1p mediates the UPR activity to resist ER stress [34]. Cell survival during Tm-induced stress requires Ire1p, which is responsible for approximately 40% of the transcriptional changes induced by Tm [4]. Ire1p disruption impairs its unfolded-protein-associated ability to respond to stress stimuli, likely causing the accumulation of unfolded proteins in the ER and stress damage [35].

Consistent with our observation, either* IRE1 *or* HAC1 *deficiency completely abolishes resistance to pharmacological ER stress responses in the long-lived deletion strains* alg12Δ *and* bst1Δ*, suggesting that* IRE1* and* HAC1* deficiency places cells at a disadvantage in the presence of ER stress responses [17]. Thus, the UPR activity was likely required for the survival of the* gas1Δ* strain in the presence of Tm-induced stress, but the specific mechanisms should be further explored.

### 3.5. The UPR Activity Exhibits No Obvious Differences between the GAS1-Deficient and Wild-Type Strains under the Tunicamycin-Induced Stress

Based on the above results, to further explore the underlying mechanism between the growth of* gas1Δ *yeast cells and UPR activity under the Tm-induced stress, we evaluated the intracellular UPR activity via an* HAC1* mRNA splicing analysis in the BY4742,* gas1Δ*, and* GAS1 OE *strains with or without Tm treatment. The results showed that, in the absence of Tm-induced stress, the* gas1Δ *strain displayed increased expression of spliced* HAC1* mRNA (47±13%), relative to the BY4742 (20±6%) and* GAS1 OE* (27±7%) strains.

**Figure 5:**
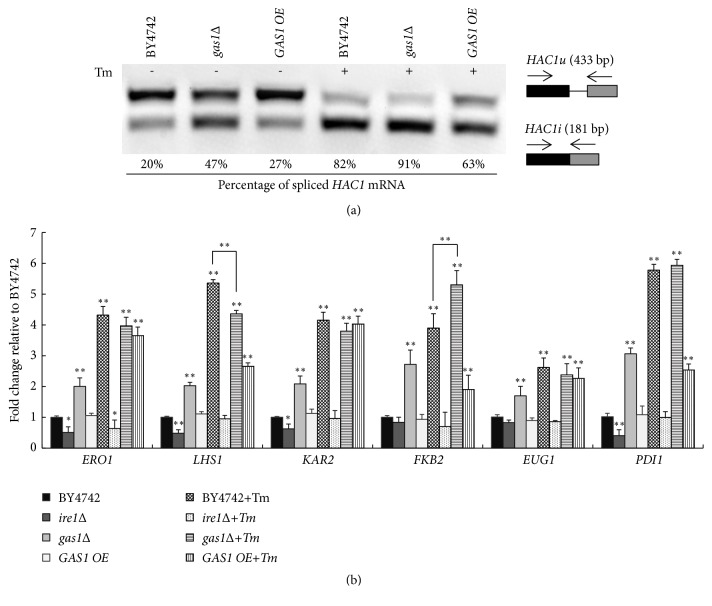
No obvious differences in UPR activity between the* GAS1*-deficient and wild-type strains under the tunicamycin-induced stress. (a)* HAC1* mRNA splicing in the mutant strains treated with or without 1 *μ*g/mL Tm for 1 h.* HAC1u *and* HAC1i* indicate the unspliced and spliced* HAC1* mRNA, respectively. (b) Relative expression of UPR target genes, such as* EUG1*,* ERO1*,* LHS1*,* KAR2*,* FKB2*, and* PDI1* in the BY4742,* gas1Δ*, and* GAS1 OE *strains. The data represent the means ± S.D. (*n* = 4). *∗ p *< 0.01* vs* BY4742; *∗∗ p *< 0.01* vs* BY4742. BY4742 was considered to be the control.

After Tm treatment, the percentage of* HAC1* mRNA splicing increased in all three strains (compared with that under the physiological conditions), and the* gas1Δ* strain displayed a slightly higher level of spliced* HAC1* mRNA (91±13%) than the BY4742 (82±15%) strain, and the* GAS1 OE* strain displayed an obviously lower level of spliced* HAC1* mRNA (63±11%) (Figure 5(a)). These data suggest that the* HAC1* mRNA splicing was not altered significantly in the* gas1Δ *strain compared with the BY4742 strain under the Tm-induced stress, but it decreased in the* GAS1 OE* strain.

Furthermore, quantitative RT-PCR was used to evaluate the transcription of several canonical UPR target genes in these mutant strains under the Tm-induced stress. Our results demonstrated that Tm treatment significantly increased the transcription of several canonical UPR target genes in the BY4742,* gas1Δ*, and* GAS1 OE *strains. The expression of most UPR target genes was not altered in the* gas1Δ *strain compared with that observed in the BY4742 strain, whereas the transcription of* LHS1* was significantly higher and that of* FKB2* was significantly lower in the* gas1Δ* strain.

However, the transcription of most of UPR target genes in the* GAS1 OE* strain showed obviously lower expression than in the BY4742 strain, such as* ERO1*,* LHS1*,* FKB2*, and* PDI1 *(Figure 5(b)). The decreased UPR target genes might not deal with the accumulated misfolded proteins within the ER induced by Tm, which ultimately overwhelmed the ER and led to decreased proliferation rate.

Based on these results, the UPR activity was not altered in the* gas1Δ *strain compared with that observed in the BY4742 strain under the Tm-induced stress, but it decreased in the* GAS1 OE* strain.

The UPR signalling pathway plays very important roles in alleviating ER stress. ER homeostasis can be restored via the UPR through the degradation of misfolded proteins, the inhibition of translation, and increased expression of chaperones that enhance the ER protein-folding capacity. Previous study showed that the* cia2Δ *yeast cells exhibited a resistance to ER stress and an enhanced UPR activity, suggesting that UPR activity could confer stress resistance to the mutant strain [36, 37]. However, in our study, no obvious differences in the UPR activity were observed between the* GAS1*-deficient and wild-type strains under the Tm-induced stress, indicating that the characterized UPR pathway may not be the main factor responsible for the observed difference in proliferation rates decreasing between the* gas1Δ *and wild-type strains under Tm-induced stress. One possibility is that the other protective signalling pathways are likely induced, such as the fact that* GAS1* deficiency triggers a set of compensatory mechanisms (the* PKC1*-*SLT2* mitogen-activated protein kinase-signalling module), maintain the cell integrity, and protect cell from injury [29–31].

Consistent with our observations, the low-UPR-activity mutant* eos1Δ *yeast cells exhibited the ER stress tolerance, with a lower* KAR2* transcription level than that observed in the wild-type strain when exposed to Tm [37], and the reason for the decreased UPR in* eos1Δ *strain may be the fact that the EOS1-deficiency could depress the inhibition of N-glycosylation caused by Tm. On the other hand, a previous study found that enhanced ER stress resistances in ribosomal protein gene deletion mutants did not depend on Hac1p, strongly suggesting that reduced translation protects against ER stress injury by a mechanism distinct from the canonical ER stress response pathway [38]. Furthermore, the folding space for unfolded proteins in the ER membrane is enlarged through a mechanism that does not require the UPR signalling pathway and reduces cell toxicity caused by ER stress [39]. Therefore, our findings and those of previous studies suggested that the UPR pathway may not be the sole pathway used to resist ER cell stress.

## 4. Conclusions 

Our results demonstrate, for the first time, that* GAS1 *deficiency shortens the lifespan of yeast cells and enhances UPR activity in the absence of Tm-induced stress. The* gas1Δ *strain exhibited the ER stress resistance under the high-Tm-concentration stress, and* IRE1* and* HAC1 *(two upstream modulators of the UPR) were required for the survival of the* GAS1 *deficiency strain when exposed to Tm. In conclusion, our findings highlight that Gas1p plays an important role in the ageing and ER stress responses in yeast.

## Figures and Tables

**Table 1:** Yeast strains used in this study.

Strain	Genotype	Source
BY4741	MATa *his3Δ1 leu2Δ0 met15Δ0 ura3Δ0*	Gift from Dr. Matt Kaeberlein
BY4742	MAT alpha* his3Δ1 leu2Δ0 lys2Δ0 ura3Δ0*	
*gas1Δ*	MAT alpha* gas1::LEU2*	This study
*GAS1OE*	MAT alpha *GAS1::pRS305-LEU2*	This study
*ire1Δ*	MAT alpha *ire1::URA3*	Previous study [13]
*hac1Δ*	MAT alpha *hac1::URA3*	Previous study [13]
*gas1Δire1Δ*	MAT alpha *gas1::pRS305-LEU2 ire1::URA3*	This study
*gas1Δhac1∆*	MAT alpha* gas1::pRS305-LEU2 hac1::URA3*	This study

**Table 2:** Nucleotide sequences of primers used for quantitative real time qPCR.

Genes	Primer pairs	Primer Sequence (5′-3′)
*PRP8*	Forward	CATGGCTGCGTCTGAAGTA
	Reverse	GGCTCAAACCCTTCCGATAG
*EUG1*	Forward	TATCAATCCACTTGCCAAACACTAC
	Reverse	ACCACTGAGTTAGAGCAACGGAA
*ERO1*	Forward	ATGGTGGTAAGCAAGCTGGTC
	Reverse	ACCGATAGAGGCATGGAAACC
*LHS1*	Forward	CCAGGTGAACAGCAGCATTATAT
	Reverse	CTATTGTAACGGGCTGAGTAGTGTC
*KAR2*	Forward	ATACGAGGGTGAAAGAGCCATG
	Reverse	TCGGATTTACCAGTTCCCTTATCT
*FKB2*	Forward	AATCGGGAACTGTATTTGACTCAA
	Reverse	TTGGAATTTGCAGCTTTCTTTT
*PDI*	Forward	CATTCCAGGGTTCCCAAGC
	Reverse	CGGATTGGACGATAACTGGAG
*GAS1*	Forward	GCTGCTGCTTTTTTTGCTGG
	Reverse	TGACAGTAGATCCGCTAGTTTCAT

## Data Availability

The data used to support the findings of this study are available from the corresponding author upon request.

## Abbreviations

ATF6: Activating transcription factor 6
ER: Endoplasmic reticulum
Gas1p: Beta-1,3-glucanosyltransferase
GPI: Glycosylphosphatidylinositol
*HAC1*: Homologous to ATF/CREB 1
IRE1: Inositol-requiring protein 1
OD: Optical density
PERK: Double-stranded RNA-activated protein kinase-like ER kinase
RLS: Replicative lifespan
*S. cerevisiae*: * Saccharomyces cerevisiae*
Tm: Tunicamycin
UPR: Unfolded protein response.

## Supplementary Materials

Supplementary Materials (1) Nucleotide sequences of primers used for vectors construction are listed in Table S1. (2) Nucleotide sequences of primers used to verify mutant strains are listed in Table S2. (3) Agarose gels of PCR products for verifying the* GAS1*-deletion,* GAS1*-overexpression, and* gas1Δire1Δ *and* gas1Δhac1Δ* strains are shown in Figures S1 and S3–S5.
