# Supplementary material for: GAS1 Deficient Enhances UPR Activity in Saccharomyces cerevisiae
Source: Biomed Res Int. 2019 Jun 2;2019:1238581. doi: 10.1155/2019/1238581 (PMC6582843; doi:10.1155/2019/1238581)
Supplement: Supplementary Materials — (1) Nucleotide sequences of primers used for vectors construction are listed in Table S1. (2) Nucleotide sequences of primers used to verify mutant strains are listed in Table S2. (3) Agarose gels of PCR products for verifying the GAS1-deletion, GAS1-overexpression, and gas1Δire1Δ and gas1Δhac1Δ strains are shown in Figures S1 and S3–S5. [file 1238581.f1.zip › Table S1.docx]

**Table S1 Nucleotide sequences of primers used for constructing the vectors**

| Vectors | Primer pairs | Primer Sequence (5'-3') | Amplicon size |
| --- | --- | --- | --- |
| pRS305-*gas1*-ko | GAS1-N-F | ATTACGCGTGGACTGTCGCATAGGGATAA (*Mlu* I) | 518 bp |
|  | GAS 1-N-R | TCGGGATCCTGTTGAGATTTAGCTGTGTT ( *Bam*H I) |  |
|  | GAS 1-C-F | CCAAAGCTTCGACACATACATAATAACTC (*Hin*d III) | 511 bp |
|  | GAS 1-C-R | ACTACGCGTCAATTGATTGAAAATAATTC (*Mlu* I) |  |
| pRS305-*GAS1-OX* | GAS1-ox-F | ATTGAGCTCTTCGAAGGAATCTTCCAACC(*Sac* I) | 2598 bp |
|  | GAS1-ox-R | TCGGGATCCTATGGAGAAAGTACATAAAT(*Bam*H I) |  |
