# Supplementary material for: GAS1 Deficient Enhances UPR Activity in Saccharomyces cerevisiae
Source: Biomed Res Int. 2019 Jun 2;2019:1238581. doi: 10.1155/2019/1238581 (PMC6582843; doi:10.1155/2019/1238581)
Supplement: Supplementary Materials — (1) Nucleotide sequences of primers used for vectors construction are listed in Table S1. (2) Nucleotide sequences of primers used to verify mutant strains are listed in Table S2. (3) Agarose gels of PCR products for verifying the GAS1-deletion, GAS1-overexpression, and gas1Δire1Δ and gas1Δhac1Δ strains are shown in Figures S1 and S3–S5. [file 1238581.f1.zip › Table S2.docx]

**Table S2 Nucleotides sequences of primers used for verifying mutant strains by PCR**

| Strains | Primer pairs | Primer Sequence (5'-3') | Amplicon size |
| --- | --- | --- | --- |
| GAS*1* deletion  (*gas1::LEU2*;* *  *gas1Δ*) | V-gas1-F | TGGTATTCCTCATACAGCCTGC | 2682 bp |
|  | V-gas1-R | GCGGTGGTAATAGCGAATAGAAT |  |
|  | Leu2-int-F | CCTCATCTGGAAGTGGGACA | 2363 bp |
|  | V-gas1-R | GCGGTGGTAATAGCGAATAGAAT |  |
| GAS*1* overexpression  (*GAS1::pRS305-LEU2*;  *GAS1 OX*) | GAS1-qPCR-F | GCTGCTGCTTTTTTTGCTGG | 160 bp/  8161 bp |
|  | GAS1-qPCR-R | TGACAGTAGATCCGCTAGTTTCAT |  |
|  | GAS1-qPCR-F | GCTGCTGCTTTTTTTGCTGG | 3473 bp |
|  | AMP-int-R | GTGACACCACGATGCCTGT |  |
|  | Leu2-int-F | CCTCATCTGGAAGTGGGACA | 2356 bp |
|  | GAS1-qPCR-R | TGACAGTAGATCCGCTAGTTTCAT |  |
| *GAS1*/*IRE1* Double-genes deletion  (*gas1:: LEU2* *ire1:: URA3*; *gas1Δire1Δ*) | Gas1-qPCR-F | GCTGCTGCTTTTTTTGCTGG | 0 bp |
|  | Gas1-qPCR-R | TGACAGTAGATCCGCTAGTTTCAT |  |
|  | Leu2-int-F | CCTCATCTGGAAGTGGGACA | 4831 bp |
|  | V-gas1-R | GCGGTGGTAATAGCGAATAGAAT |  |
|  | V-gas1-F | TGGTATTCCTCATACAGCCTGC | 4905 bp |
|  | Amp-int-R | GTGACACCACGATGCCTGT |  |
|  | Ire1-jd-F | AAGGCGGCAGATAGTGGAA | 0 bp |
|  | Ire1-jd-R | AGAATTGGCAGCCCTACGTC |  |
|  | URA3-int-F | GGAACCTAGAGGCCTTTTGATGT | 651 bp |
|  | V-Ire1-R | ATGTCTGTCGGGTAGTTTATGTAGG |  |
|  | V-Ire1-F | CTTCCCCACGTCCATTATCACTT | 1549 bp |
|  | V-Ire1-R | ATGTCTGTCGGGTAGTTTATGTAGG |  |
| *GAS1*/*HAC1*  Double-genes deletion  (*gas1:: LEU2*  *hac1:: URA3*;  *gas1Δhac1Δ*) | Gas1-qPCR-F | GCTGCTGCTTTTTTTGCTGG | 0 bp |
|  | Gas1-qPCR-R | GACAGTAGATCCGCTAGTTTCAT |  |
|  | Leu2-int-F | CCTCATCTGGAAGTGGGACA | 4831 bp |
|  | V-gas1-R | GCGGTGGTAATAGCGAATAGAAT |  |
|  | V-gas1-F | CCTCATCTGGAAGTGGGACA | 4905 bp |
|  | Amp-int-R | GTGACACCACGATGCCTGT |  |
|  | Hac1-jd-F | TCCAGCCGTGATTACGATGA | 0 bp |
|  | Hac1-jd-R | AGCAAGCCGTCCATTTCTTAGTA |  |
|  | URA3-int-F | GGAACCTAGAGGCCTTTTGATGT | 761 bp |
|  | V-hac1-R | CTGCCCAGTCGCCACAAA |  |
|  | V-hac1-F | GGCAAAGTGGCTCAGCATTA | 1657 bp |
|  | V-hac1-R | CTGCCCAGTCGCCACAAA |  |
