# Supplementary material for: GAS1 Deficient Enhances UPR Activity in Saccharomyces cerevisiae
Source: Biomed Res Int. 2019 Jun 2;2019:1238581. doi: 10.1155/2019/1238581 (PMC6582843; doi:10.1155/2019/1238581)
Supplement: Supplementary Materials — (1) Nucleotide sequences of primers used for vectors construction are listed in Table S1. (2) Nucleotide sequences of primers used to verify mutant strains are listed in Table S2. (3) Agarose gels of PCR products for verifying the GAS1-deletion, GAS1-overexpression, and gas1Δire1Δ and gas1Δhac1Δ strains are shown in Figures S1 and S3–S5. [file 1238581.f1.zip › Figures S1-S5.doc]

**Supplementary Figure: 1** Verification of the *GAS1* deletion strain by PCR.

PCR products of the genomic DNA of *gas1Δ* strain containing the LEU2 selective marker. The 2363 bp band in lane 1 was amplified with Leu2-int-F and V-gas1-R primers; and the 2683 bp band in lane 2 was amplified with V-gas1-F and V-gas1-R primers. M represents DNA marker.

**Supplementary Figure: 2** A schematic of the pRS305-*GAS1*-OE plasmid and construction of the *GAS1 OE* strain. **a**. Primers for amplifying the *GAS1*-containing fragment which was subsequently integrated into pRS305. In addition to the entire coding region of *GAS1*, ～609 nucleotides of the upstream sequence and ～308 nucleotides of the downstream sequence were amplified. **b**. Construction of the *GAS1-OE* strain by transforming the linearized pRS305-*GAS1*-OE plasmid into BY4742. Note that after successful integration, a single endogenous copy of *GAS1* has been replaced by two copies of *GAS1*. This panel also illustrates the location of primers used to confirm the successful integration of both copies of *GAS1* in the final strain.

**Supplementary Figure:3** Verification of the *GAS1* overexpression strain by PCR.

PCR products of the genomic DNA of *GAS1 OE* strain containing the Leu2 selective marker. The 160 bp and 8161 bp bands in lane 1 were amplified with GAS1-qPCR-F and GAS1-qPCR-R primers; the 3473 bp band in lane 2 was amplified with GAS1-qPCR-F and Amp-int-R primers; and the 2356 bp band in lane 3 was amplified with Leu2-int-F and GAS1-qPCR-R primers. M represents DNA marker.

**Supplementary Figure: 4** Verification of the *gas1Δire1Δ* strain by PCR.

PCR products of the genomic DNA of *gas1Δire1Δ* strain. The 0 bp in lane 1 was amplified with GAS1-qPCR-F and GAS1-qPCR-R primers; the 4381 bp in lane 2 was amplified with Leu2-int-F and V-gas1-R primers; the 4905bp in lane 3 was amplified with V-gas1-F and Amp-int-R primers; the 0 bp in lane 4 was amplified with Ire1-jd-F and Ire1-jd-R primers; the 651 bp in lane 5 was amplified with URA3-int-F and V-Ire1-R primers; and the 1549 bp in lane 6 was amplified with V-Ire1-F and V-Ire1-R primers. M represents DNA marker.

**Supplementary Figure: 5** Verification of the *gas1Δhac1Δ* strain by PCR.

PCR products of the genomic DNA of *gas1Δire1Δ* strain. The 0 bp in lane 1 was amplified with GAS1-qPCR-F and GAS1-qPCR-R primers; the 4381 bp in lane 2 was amplified with Leu2-int-F and V-gas1-R primers; the 4905bp in lane 3 was amplified with V-gas1-F and Amp-int-R primers; the 0 bp in lane 4 was amplified with Hac1-jd-F and Hac1-jd-R primers; the 761 bp in lane 5 was amplified with URA3-int-F and V- Hac1-R primers; and the 1657 bp in lane 6 was amplified with V- Hac1-F and V-Hac1-R primers. M represents DNA marker.
